# New Sulfur-Containing Polyarsenicals from the New Caledonian Sponge *Echinochalina bargibanti*

**DOI:** 10.3390/md16100382

**Published:** 2018-10-11

**Authors:** Petri Tähtinen, Graziano Guella, Giacomo Saielli, Cécile Debitus, Edouard Hnawia, Ines Mancini

**Affiliations:** 1 Department of Chemistry, University of Turku, Vatselankatu 2, 20014 Turku, Finland; petri.tahtinen@utu.fi; 2 Laboratorio di Chimica Bioorganica, Dipartimento di Fisica, Università di Trento, Via Sommarive 14, I-38123 Trento, Italy; graziano.guella@unitn.it; 3 Istituto CNR per la Tecnologia delle Membrane, Unità di Padova, and Dipartimento di Scienze Chimiche, Università di Padova, Via Marzolo, 1-35131 Padova, Italy; giacomo.saielli@unipd.it; 4 LEMAR, IRD, UBO, CNRS, IFREMER, IUEM, 29280 Plouzané, France; cecile.debitus@ird.fr; 5 Laboratoire Insulaire du Vivant et de l’Environnement, Université de la Nouvelle-Calédonie: EA 4243 BP 11106, 98802 Nouméa, Nouvelle-Calédonie, France; edouard.hnawia@ird.fr

**Keywords:** antibacterial, arsenical, calculated NMR spectrum, density functional theory, natural products, NMR spectroscopy, structure elucidation, sulfur metabolite

## Abstract

Arsenicin A (C_3_H_6_As_4_O_3_) was isolated from the New Caledonian poecilosclerid sponge *Echinochalina bargibanti*, and described as the first natural organic polyarsenic compound. Further bioguided fractionation of the extracts of this sponge led us to isolate the first sulfur-containing organic polyarsenicals ever found in Nature. These metabolites, called arsenicin B and arsenicin C, are built on a noradamantane-type framework that is characterized by an unusual As–As bonding. Extensive NMR measurements, in combination with mass spectra, enabled the assignment of the structure for arsenicin B (C_3_H_6_As_4_S_2_) as **2**. The scarcity of arsenicin C and its intrinsic chemical instability only allowed the collection of partial spectral data, which prevented the full structural definition. After the extensive computational testing of several putative structures, structure **3** was inferred for arsenicin C (C_3_H_6_As_4_OS) by comparing the experimental and density functional theory (DFT)-calculated ^1^H and ^13^C NMR spectra. Finally, the absolute configurations of **2** and **3** were determined with a combined use of experimental and time-dependent (TD)-DFT calculated electronic circular dichroism (ECD) spectra and observed specific rotations. These findings pose great challenges for the investigation of the biosynthesis of these metabolites and the cycle of arsenic in Nature. Arsenicins B and C showed strong antimicrobial activities, especially against *S. aureus*, which is comparable to the reference compound gentamycin.

## 1. Introduction

Inorganic arsenic compounds are ubiquitous on earth and in the atmosphere. Natural organoarsenicals are also found, originating from the conversion of inorganic arsenic forms by a wide variety of marine organisms and including volatile, non-volatile, and water-soluble polar metabolites [1]. Studies have revealed the high affinity of arsenic for sulfur in both natural and synthetic processes [2]. The essential roles played by thiol groups in the metabolic pathways of arsenic involve the ability of this element to bind thiol groups of sulfur-rich peptides (i.e., glutathione) and proteins, resulting in compromised protein folding [3,4,5,6,7]. Arsenic biotransformations are catalyzed by enzymes that are present in diverse environmental organisms, and are coupled to the biogeochemical cycles of some other elements, including sulfur [8].

Nonetheless, the knowledge of natural sulfurated organoarsenic compounds remains scanty, and is limited to thioarsenic acid, which is involved in the metabolism of cod-liver arsenolipids in humans [9], 2-dimethylarsinothioyl acetic acid as the first mammalian thio-organoarsenate [10], and thio-arsenosugars present in extracts of marine organisms [11] such as mussels [12], giant clam tissues [13], and shellfish [14].

**Figure 1:**
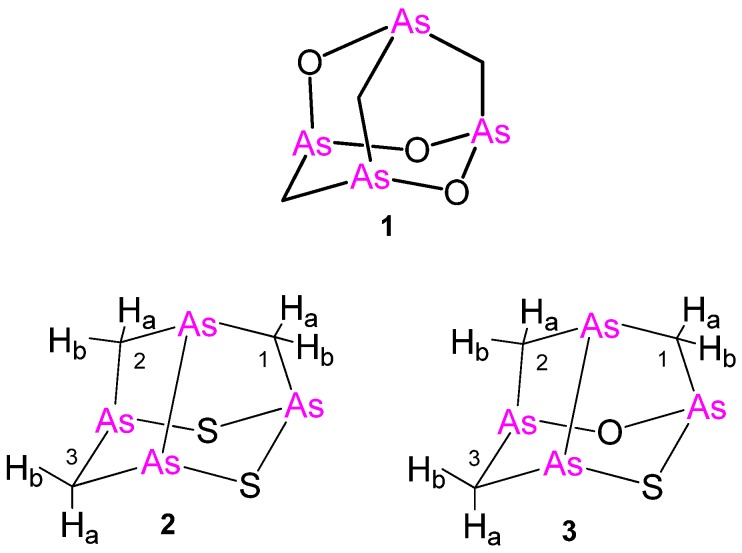
Molecular structures of arsenicin A (C_3_H_6_As_4_O_3_) (**1**) and the new arsenicin B (**2**) and arsenicin C (**3**). Arbitrary numbering is for convenience.

All of these compounds share the characteristic of containing a single arsenic atom per molecule. However, this structural feature has been recently surpassed by the isolation of arsenicin A (C_3_H_6_As_4_O_3_) from the poecilosclerid sponge *Echinochalina bargibanti* from the northeastern coast of New Caledonia. Arsenicin A, the first example of a polyarsenical of natural origin, was structurally assigned by extensive NMR and IR spectra measurements and *ab initio* calculations to have an adamantane-type backbone (**1**, Figure 1), and its structure was confirmed by the synthesis of an analogue [15]. Racemic arsenicin A was later synthesized, and its crystal structure was described [16]. In subsequent cytotoxicity evaluation on some human carcinoma cell lines, it proved to be very efficient [17]. Inspired by these findings a further lead optimization was carried out by widening the molecular diversity of arsenicin A.

Some related compounds to arsenicin A were produced by an efficient one-pot microwave-assisted synthesis starting from arsenic (III) oxide, which reduces the risk associated with the handling of these dangerous chemicals. The in vitro cytotoxicity screening of these compounds on a full panel of cancer cell lines at the National Cancer Institute (NCI-USA) indicated that the most lipophilic arsenicin A analogue is responsible for the best growth inhibition of both leukemia and solid tumor cell lines, with IC_50_ values lower than arsenic trioxide used as a control test compound [18].

Arsenic is the epitome of toxicity and, indeed, all of the arsenic compounds are toxic to some degree. Nevertheless, some arsenicals have been used as therapeutic agents. Significant examples include the synthetic drug salvarsan, which has been used to treat syphilis and trypanosomiasis, which is also known as the sleeping sickness, and arsenic trioxide, which has been exploited in traditional Chinese medicine for a long time. It was also approved for the treatment of acute promyelocytic leukemia by the Food and Drug Administration (FDA) in 2000 and is currently studied for the treatment of solid tumors [19]. In the context of the results obtained so far with both arsenicin A and its synthetic analogues, we expect that new related arsenicals may contribute to promising perspectives in the development of novel arsenical drugs. This, in turn, makes their molecular structures worthy of significant consideration.

A careful examination of the minor metabolites isolated by extracts of the New Caledonian sponge *E. bargibanti* provided further advances on polyarsenicals. We describe here the first natural polyarsenicals built on a noradamantane framework, containing unprecedented arsenic–sulfur bonds and a very unusual arsenic–arsenic bond.

## 2. Results and Discussion

### 2.1. Isolation and Experimental Structural Characterization of Arsenicins B and C

Raw dichloromethane extracts of the residue from ethanol treatment of lyophilized *E. bargibanti* were subjected to bioassay-guided silica flash chromatography (FC). Bioactive fractions from antibacterial and antifungal assays were freed from sterols and carotenoids by reverse-phase FC. Preparative HPLC purification performed under careful handling provided, besides the known arsenicin A (**1**) [15], also low amounts of less polar sulfurated tetraarsenicals arsenicin B (**2**) and arsenicin C (**3**) (Figure 1). The 254-nm absorption wavelength utilized for detection in HPLC purification was in agreement with that used for arsenicin A. In fact, despite the absence of an obvious chromophore, these metabolites are UV absorbent at relatively high wavelengths similar to their synthetic analogues [18], which is a feature that has been elegantly simulated by time-dependent density functional theory (DFT) calculations [20].

In analogy with arsenicin A (**1**) [15], the mass defect electron impact-mass spectrometry (EI–MS) signals of arsenicin B hinted about the presence of arsenic atoms, whereas the isotopic cluster at *m*/*z* 406 accounted for the presence of three carbon and two sulfur atoms (Figure S1). The molecular composition C_3_H_6_As_4_S_2_ was confirmed by high resolution EI–MS experiments performed on the molecular peak at *m*/*z* 405.6778 ± 0.002 (calcd. 405.6774). The fragmentation pattern, in analogy with that of arsenicin A [15], showed a fragment ion at *m*/*z* 360, which was derived by a loss of one thioformaldehyde molecule from the molecular ion, and the signals at *m*/*z* 300, 225, and 150 due to the formation of tetrameric, trimeric, and dimeric arsenic ion species, respectively.

Unlike arsenicin A, arsenicin B proved to be quite reactive in the mass spectrometric chamber. In fact, during the acquisition of a set of spectra, a peak at *m*/*z* 422 (ca. 5% intensity with respect to the molecular ion) arose, while the intensity of the signal at *m*/*z* 360 peak, corresponding to (M-CH_2_S)^+^•, decreased concurrently. The proneness of arsenicin B to oxidation was also evident when performing atmospheric pressure chemical ionization (APCI)-MS experiments, where O_2_ is required in the ionization process. In these experiments, beside the protonated molecular ion [M + H]^+^ at *m*/*z* 407, which was detected as the most intense signal, adduct ions appeared at *m*/*z* 423 and *m*/*z* 439. The latter signal can be attributed to a [MH(MeOH)]^+^ ion, since methanol was used as the solvent for direct injection of the sample into the source. In accordance, the intensity of this signal increased upon lowering the applied voltage. Tandem fragmentation (+)APCI-MS/MS experiments, carried out on the *m*/*z* 407 signal, revealed the loss of a CH_2_S fragment. Envisaging sulfur in the place of oxygen atoms, this behavior recalls the fragmentation that is observed for both arsenicin A and its synthetic model compound, where the loss of CH_2_O and CH_3_CHO, respectively, was observed [15], as well as that observed for the synthetic arsenicin A-related molecules that have been recently reported [18].

The ^1^H-NMR spectrum of arsenicin B in CDCl_3_ showed signals for six magnetically non-equivalent protons. The signals of geminally coupled protons, which recall those observed for arsenicin A [15], are correlated to each other accounting for three methylene units, as supported by the observed carbon multiplicities and the results of HSQC and HMBC experiments (Figure S2, Table 1).

The observation that all of the hydrogen and carbon atoms give rise to individual signals hinted at a structure with lower symmetry than arsenicin A, which limits the constraints on any proposed atom connectivity. Preliminary assignments resulted in the connectivities shown in Table 1, and were supported by the following observations: (a) the three ^13^C signals belong to the methylene groups according to the APT experiments; (b) the CH_2_ groups cannot be close neighbors, since no vicinal couplings were observed, implying that the largest couplings arise from geminal protons; c) the CH_2_ groups must be suitably positioned with respect to one another to account for the long-range couplings that have been observed. The molecular composition of arsenicin B, its optical activity, and the NMR spectra strongly suggested the asymmetric noradamantane-type skeleton **2** (Figure 1), showing an As–As bond related to uzonite (As_4_S_5_). This structure was supported by the HMBC correlations observed for the δ_H_ 2.34 (1-Hb) with the δ_C_ 27.40 C-2, as well as by a W-coupling between 2-Ha and 3-Ha (*J* = 1.7 ppm).

It is noteworthy that arsenicin B corresponds to one of the four sulfur derivatives obtained when synthetic arsenicin A was treated with an aqueous solution of sodium sulfide in benzene [21]. The NMR data of the metabolite isolated from *E. bargibanti* extract are practically superimposable to those reported for the racemic (R*As, S*As, R*As, R*As)-dihydro-3H-2,6-epithio(1,2,4)triarsolo-(1,2-b)(1,2,3,5) thiatriarsole, which was structurally determined by X-ray diffraction after racemate resolution by chiral column chromatography.

The structural formula C_3_H_6_As_4_OS for the optically active arsenicin C was deduced from a high resolution EI–MS measurement that revealed a molecular peak at *m*/*z* 389.7059 ± 0.007 (cald. 389.7003) with an isotopic pattern that is in good agreement with this elemental composition. Similarly to arsenicin B, arsenicin C proved to be liable to oxidation under EI–MS and APCI-MS conditions, and it was found to form adduct ions with methanol during APCI ionization. The loss of CH_2_S was observed in the EI–MS spectrum leading to an intense peak at *m*/*z* 344 (corresponding to C_2_H_4_As_4_O^+^• from high resolution (HR)–MS experiments). Fragments at *m*/*z* 361 and 345, via the APCI-MS experiments in positive ion mode, were attributed to the loss of CH_2_O and CH_2_S from the molecular ion respectively (Figure S3).

The ^1^H-NMR spectrum for arsenicin C resembled that of arsenicin B, with six signals corresponding to three methylene units. This was supported by the HSQC correlations, with three carbon atoms appearing as triplets in the APT experiments (Table 1). HMBC experiments revealed the correlation of 2-Ha at δ_H_ 3.50 with C-1 at δ_C_ 45.14; W couplings were detected among the following couples: 3-Ha/2-Ha (*J* = 1.9 Hz) and 1-Hb/2-Hb (*J* = 1.7 Hz), whilst nuclear Overhauser enhancement (nOe) effects were found only within geminal pairs. These NMR data are in agreement with the dihedral angles and the interatomic distances of all of the DFT minimized structures with C1 symmetry derived by swapping around the position of oxygen and sulfur atoms (**C1**–**C3** structures, as discussed in the next section), and not allowing to discriminate between them.

Infrared and Raman spectroscopy had shown to be very useful tools in the structure elucidation of arsenicin A and related synthetic polyarsenicals, by the comparison of the experimental and DFT-calculated spectra for a series of plausible candidate structures [15,18,22]. In particular, the vibrational analysis based on DFT-minimized geometries showed significant differences in the calculated low-frequency IR absorption bands for structures **C1**–**C3**. Unfortunately, this potential discrimination criterion could not be applied because of the low amount and the labile nature of arsenicin C, which proved to degrade during the sample preparation for IR spectral measurements.

### 2.2. Computational NMR Analysis

The structure of natural arsenicin A was assigned without the decisive contribution of NMR analysis, because (i) the simple spin system and spatial arrangement of the molecule (essentially isolated methylene groups) severely limited the connectivity information that could be extracted from the experimental NMR spectra; (ii) the chemical shifts could hardly be compared to the known data owing to the dearth of such reference data; and (iii) unattainable investigation in this case of the most natural NMR probe, ^75^As, (I = 3/2, 100% natural abundance), owing to its high quadrupole moment, which leads to extremely wide lines in all of the common bonding geometries of As(III). However, the known predictive power of the DFT NMR computational method could be effectively applied to arsenicin A-like systems, since they are relatively small, rigid, and non-polar molecules, although they contain a set of heavy As atoms. This method proved to be effective in predicting the NMR spectral patterns for arsenicin A, providing further structural validation [23]. Moreover, the experimental ^1^HNMR data recently obtained for the first synthesized isomer of arsenicin A (=2,4,10-trioxa-1,3,5,7-tetraarsaadamantane) [18] resulted in agreement with the chemical shifts that have been previously calculated for a trial structure that was considered in computational studies [23].

In the light of these validation steps on arsenicin A and its analogues, we reckoned that the NMR computational elucidation of arsenicin B and arsenicin C would provide a valuable contribution, especially helping to discriminate among equally compatible structures based on the NMR experimental data acquired for arsenicin C. In fact, similar to arsenicin A, these minor sulfurated metabolites also exhibit simple NMR spectra from which molecular connectivities can hardly be extracted without further assumptions concerning the chemical shift values.

#### 2.2.1. Arsenicin B

**Figure 2:**
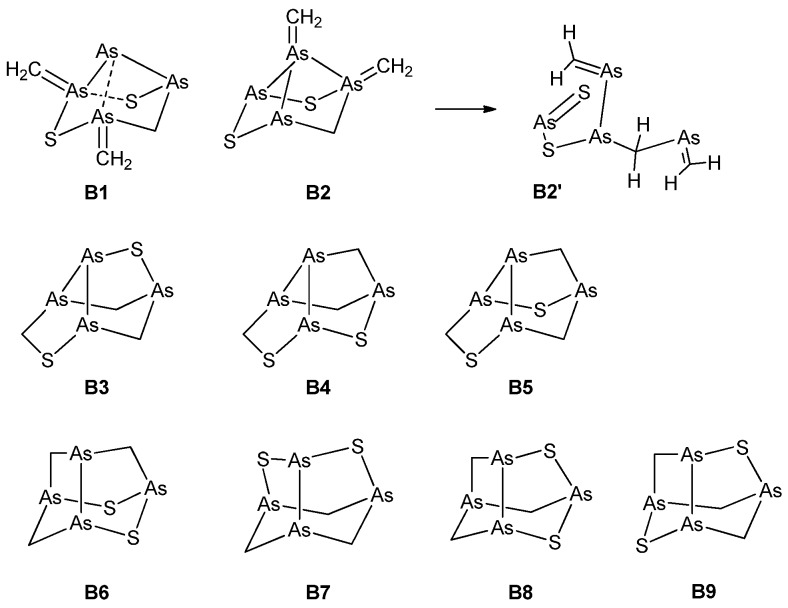
Trial structures for arsenicin B. **B1** and **B2** feature four-membered and six-membered-rings; **B3**–**B5** feature a seven-membered ring; **B6**–**B9** feauture only five-membered and six-membered rings. Structure **B2** is computationally unstable, and evolves to **B2’** upon optimization (see text). The bonds indicated by dashed lines in **B1** are not fully occupied, as shown by a natural bond orbital (NBO) analysis calculation.

With the evidence gathered from the experiments as illustrated above, various trial structures were considered for arsenicin B and their calculated chemical shifts compared with the experimental ones. The candidate structures herein evaluated fall into three categories, embodying four-membered rings, or seven-membered rings, or, finally, both five-membered and six-membered rings. The structures are shown in Figure 2 and, in addition to the comparison of chemical shifts, were also screened with regard to their energies.

Structures with four-membered rings (**B1**, **B2**) require the presence of ylide groups (As=CH_2_). While **B1** represents a minimum on the potential energy surface (PES), **B2** does not, as its optimization leads to the open-chain structure **B2’**. As far as structures incorporating a seven-membered ring are concerned, it must be said that although **B3**–**B5** (Figure 2) looked promising, no good correlation could be obtained. In fact, computed ^13^C chemical shifts were in the range of 40–72 ppm, and did not support any of these structures.

Likewise, as for **B1** and **B2**, the structures **B3**–**B5** also exhibited exceedingly high energies (ΔE = 200–300 kcal/mol) with respect to **B6**–**B9** (Supplementary Table S1). In view of their high energies, their geometry and electronic structure were not further characterized. Obviously, such low thermodynamic stabilities strongly argue against the formation of all of these structures.

Structures incorporating only five-membered and six-membered rings (**B6**–**B9**) were much more thermodynamically stable, all lying within 1.3 kcal/mol from each other. Each of them contained an As–As bond within a noradamantane arrangement (Figure 2). Although the most stable structure is **B9**, only the **B6** structure has the required C1 symmetry. Therefore, the results for **B6** were analyzed in detail. The comparison between the experimental and calculated chemical shifts of **B6** (Table 2, Figure S4) highlights the large (ca. 7 ppm) spin-orbit contributions to σ (^13^C), arising presumably from the adjacent As–As bond. Its inclusion (method C, defined as described in Section 3.4) leads to slightly lower MAEs (mean absolute errors). The MAE of relativistic ^1^H chemical shifts was extremely low, indicating that, together with the good linear correlation with the experimental values, the accuracy of the calculations is very good. The calculated ^13^C chemical shifts are systematically larger than the experimental values by about 15 ppm; nevertheless, the correlation between them is excellent in each case.

The DFT calculated ^75^As nuclear quadrupolar coupling constants (NQCC) for the four arsenic nuclei in **B6** are high, ranging between −185 and −149 MHz, and together with their computed asymmetry parameters (η = 0.10–0.68, method C, defined as described in Section 3.4). They lead to very broad linewidths of ^75^As NMR resonances, and thus rule out the possibility to utilize ^75^As as a viable probe in the structural determinations [23].

Also, the experimental and calculated *J* (H,H) values for arsenicin B were compared. As a result, all of the major geminal couplings were correctly modeled and led to a correct sequence and pattern. The calculations also confirmed the negative sign of the geminal couplings. However, the observed smaller coupling (1.7 Hz) was never matched to the largest computational value (Table S2). On the other hand, the calculations predicted small long-range couplings between almost all of the protons, but in general, their magnitude was at the limits of computational accuracy and experimental detection. A comparison between the experimental and calculated ^1^H spectra of structure **B6** is given in Supplementary Figure S5. After the extensive testing of several putative structures of the disulfurated arsenicin B, the computational study found agreement for structure **B6**, which is the one assigned by experimental data and supported by the X-ray analyzed synthetic compound [21].

Finally, the absolute configuration of arsenicin B was determined by comparing its experimental and time-dependent density functional theory (TD-DFT) calculated electronic circular dichroism (ECD) spectra (Figure S6). The theoretical spectrum calculated at the B3LYP/6-311+G(3df,2pd) level of theory for the (1*R*,3*S*,5*R*,7*R*)-enantiomer showed negative Cotton effects at 293 nm and 218 nm, and a positive one at 262 nm, which are in agreement with the observed Cotton effects for arsenicin B, and thus confirm the indicated absolute configuration (corresponding to (*R* As_1,2_, *S* As_3,S_, *R* As_2,3_, *R* As_S,1_) based on the numbering indicated in Figure 1). The utilized level of theory was previously found to be satisfactory for the computation of electronic properties for arsenicin A [20].

#### 2.2.2. Arsenicin C

**Figure 3:**
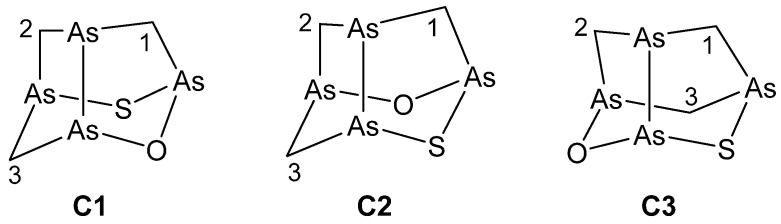
Trial structures considered for arsenicin C in NMR computational analysis.

On the basis of the results for arsenicin B, only the three trial structures **C1**–**C3** of the C1 symmetry group (Figure 3) were considered for arsenicin C.

According to the computed total energies (Table 3), **C2** is the most stable structure. Its chemical shifts calculated with all three methods showed the best correlations with the experimental values (Table 4, Figure S7). Structure **C3** was clearly incorrect, and poorer correlations were also observed for structure **C1**, although the MAEs were better for it than for **C2** (Table 4).

However, the linear fitting parameters obtained with each method for **C2** coincided with the parameters obtained for **B6** (i.e., the data of **C2** and **B6** fell on the same correlation line). Therefore, it could be concluded that **C2** is the best candidate for the structure of arsenicin C.

Similarly to arsenicin B, the ^75^As NQCC values for arsenicin C were high, ranging between −222 and +205 MHz together with =0.10–0.95 (method C), rendering ^75^As again a useless NMR nucleus due to expected very broad line widths [23].

Coupling constants were calculated for candidate structures **C1** and **C2** (Table 5). Again, the couplings calculated with the pcJ-2 basis set (method B) gave the best estimates for the ^2^*J* values. The calculated coupling constants for **C2** correlated only marginally better with the experimental ones compared to those calculated for **C1**. However, given the closely similar backbones, it is not surprising that the most relevant ^2^*J* couplings are all very similar. The computations predicted the observed small ^4^*J*(H1b,H2b) and ^4^*J*(H2a,H3a) for **C2**, but also other long-range couplings that were not observed. However, once again, the magnitudes of these couplings were mostly within the ^1^H-NMR natural linewidths, as well as within the limits of computational accuracy. Therefore, they were neglected.

**Figure 4:**
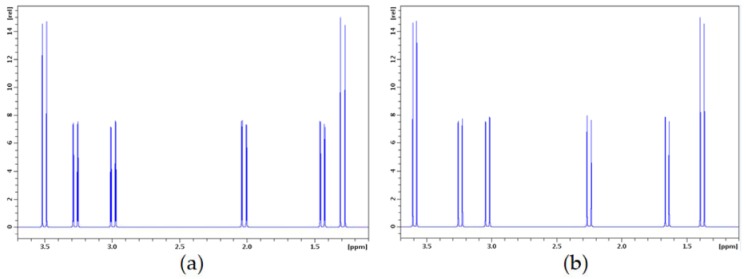
Experimental (**a**) and calculated (**b**) ^1^H spectra of arsenicin C and structure **C2**, respectively. Spectrum recorded in CDCl_3_ at 400 MHz.Chemical shifts calculated with method (C), couplings with method (B). Simulations at 400 MHz.

In conclusion, a good agreement was found between the experimental and calculated ^1^H spectra of structure **C2**, as illustrated in Figure 4. On this basis, structure **3** (=**C2**) for arsenicin C was assigned. Furthermore, as the observed specific rotation for arsenicin C had the same sign as that for arsenicin B, and they are also very similar in magnitude, it is highly plausible that they also possess the same skeleton chirality, as determined above for arsenicin B.

### 2.3. Biological Activity

Antimicrobial tests with human pathogenic clinical strains of Gram-positive *Staphylococcus aureus*, Gram-negative *Escherichia coli*, and of the fungi *Candida albicans*, were carried out using the disk (six-mm diameter) diffusion assay [24] loaded with concentrations of the pure metabolite of 10 µg/disc, five µg/disc and one µg/disc. Arsenicins A–C showed strong antifungal activity and extremely potent antimicrobial activity compared to that of the antibiotic gentamycin, as shown in Table 6.

It was evident that arsenicin B and arsenicin C were only slightly less active than arsenicin A [15]. They proved to be especially effective against *S. aureus* which, having become resistant to clinical antibiotics, is responsible for common devastating infections in humans worldwide. In particular, the data obtained at 10 µg/disc for arsenicins A–C and gentamycin indicated that the activity decreased with the increased presence of sulfur atoms. The medical use of arsenic compounds gained popularity in the last centuries, especially in Chinese medicine. More recently, their use as antimicrobial agents has been limited to the treatment of syphilis with the synthetic drug salvarsan. On the other hand, the emergence of multiple drug-resistant strains of bacteria due to the indiscriminate use of antibiotics has induced an urgent need of developing additional strategies to treat bacterial infections. It is also to note that the synthetic polyarsenical with the composition C_3_H_6_As_4_S displayed values of proliferation inhibition that were higher than arsenicin A on ovarian and breast cancer cell lines [21]. These findings may open new perspectives in considering polyarsenicals with one or more sulfur atoms as potential applications in drug development.

## 3. Materials and Methods

### 3.1. General Experimental Procedures

Polarimetric data were recorded with a Jasco DIP-181 polarimeter; [α]_D_ values are given in 10^−1^ deg cm^2^ g^−1^. UV spectra were obtained with a Perkin-Elmer Lambda-3 spectrophotometer. Cotton effects were observed in a circular dichroic (CD) spectrum, recorded with a Jasco J-710 spectropolarimeter. Nuclear magnetic resonance (NMR) spectra were acquired with an Avance 400 Bruker spectrometer and (when stated) a Varian XL 300 spectrometer. Avance: ^1^H at 400 MHz in CDCl_3_; XL 300 ^13^C at 75.43 MHz. δ values in ppm rel. to SiMe_4_ (=0 ppm) and *J* values in Hz; ^1^H,^1^H correlations from standard and long-range correlated spectroscopy (COSY) experiments and selective decoupling irradiations; ^1^H,^13^C assignments from heteronuclear single quantum correlation (HSQC) and heteronuclear multiple bond correlation (HMBC) experiments; nuclear Overhauser enhancement (nOe) data from both differential one-dimensional (1D) nOe, obtained with 5 s of preirradiation, and bidimensional NOESY experiments. ^13^C NMR data from heteronuclear bidimensional experiments at 400 MHz, or 1D measurements (XL 300) at 75.43 MHz. Multiplicity from APT experiments. Electron impact (EI)–MS (*m*/*z*; rel. %) and high-resolution (HR)–EI–MS spectra were recorded with a Kratos MS80 mass spectrometer equipped with home-built computerized acquisition software. APCI-MS and tandem (MS/MS)^n^ were acquired with a Bruker Esquire–LC^TM^ mass spectrometer equipped with an atmosphere pressure chemical ionization ion source used in positive ion mode. Samples were injected into the source as a methanol solution.

### 3.2. Collection and Isolation

The sponge (R1858/881m) was collected along the northeastern coast of New Caledonia at 18–25 m depth during the Substances Marines d’Intérêt Biologique (SMIB) program [25]. The sponge (6.2 kg moist) was immediately frozen and then freeze-dried (480 g dry weight). A small amount of freeze-dried sponge was extracted (EtOH), evaporated, CH_2_Cl_2_/H_2_O partitioned, and the residue from the evaporation of the organic phase was subjected to gradient flash chromatography (Si-60, *n*-hexane/AcOEt), bioguided by both *S. aureus* and *Candida albicans* activities. This procedure was then carried out on the active fractions from the whole freeze-dried material. The CH_2_Cl_2_ extract (20 g) was subjected to flash chromatography, collecting 26 fractions of 0.1-L each. Combined fractions 6–15 were subjected to reversed-phase flash chromatography (RP-18, MeCN/H_2_O), setting free the organoarsenicals (UV spots) from the sterols and carotenoids. The solvent, MeCN, was evaporated at room temperature, and the remaining aqueous residue was extracted with AcOEt and evaporated. The residue was subjected to preparative HPLC purification (Lichrosorb CN, 7 μm, 254 nm) with *n*-hexane/AcOEt 96:4 to give, in the order of elution, arsenicin B (t_R_ 6.7 min, 2.0 mg, 0.0004% on freeze-dried sponge), arsenicin C (t_R_ 8.0 min 1.2 mg, 0.0002%), and arsenicin A (5.2 mg, 0.001%). The two minor compounds proved to be labile under the routine aerial workup, so that their isolation from elution by preparative HPLC was repeated under a nitrogen atmosphere in order to minimize oxidative degradation.

### 3.3. Data of Arsenicin B and Arsenicin C

*Arsenicin B (**2**)*. White powder. ${\left[\alpha \right]}_{D}^{20}$ = −16° (c 0.4 in CHCl_3_). λ_max_ (MeOH)/nm 275 (ε/dm^3^ mol^−1^ cm^−1^ 660), 245 (2800), and 203 (13,000). CD (MeOH): Δε(λ) = −1.5 (286), +0.8 (251), −3.2 (216). NMR data: in Table 1. EI–MS: *m*/*z* 408 (9, [M + 2]^+^•), 407 (4, [M + 1]^+^•), 406 (100, M^+^•), 360 (38, [M-CH_2_ S]^+^), 319 (3), 317 (32, C_2_H_4_As_3_S_2_^+^), 300 (2), 299 (19, C_3_ H_6_ As_3_S^+^), 285 (11), 271 (15), 257 (14, As_3_S^+^), 253 (9), 239 (9), 225 (16, As_3_^+^), 182 (11), 163 (13), 150 (8), 121 (5), 107(40, AsS^+^), 89 (12, AsCH_2_^+^), 75 (5, As^+^); HR–EI–MS: *m*/*z* 405.6778 ± 0.001 (C_3_H_6_As_4_ S_2_^+^•; calcd. 405.6774); 359.6905 ± 0.001 (C_2_H_4_As_4_S^+^•; calcd. 359.6898); 106.8942 ± 0.001 (AsS; calcd. 106.8937). APCI-MS (positive ion mode): *m*/*z* 455 [(M + O + H + MeOH)^+^], 439 [(M + H + MeOH)^+^], 423 [(M + O + H)^+^], 407 [(M + H)^+^]; APCI-MS/MS (407): *m*/*z* 361 [(M + H -CH_2_S)^+^].

*Arsenicin C (**3**)*. White powder. ${\left[\alpha \right]}_{D}^{20}$ = −20° (c 1.0 in CHCl_3_). NMR data: in Table 1. EI–MS: *m*/*z* 392 (4, [M + 2]^+^•, 391 (3, [M + 1]^+^•), 390 (79, M^+^•), 360 (16, [M -CH_2_O]^+^•), 344 (25, [M -CH_2_S]^+^•), 301 (21, [M -CH_2_As]^+^), 300 (2), 299 (9, [M -As O]^+^), 283 (2, [M -AsS]^+^), 253 (9), 225 (14), 163 (19), 107 (24, AsS^+^), 91 (15, AsO^+^), 89 (13, AsCH_2_^+^), 75 (3, As^+^). HR–EI–MS: *m*/*z* 389.7059 ± 0.007 (C_3_H_6_As_4_OS^+^•; calcd. 389.7003); 343.7116 ± 0.001 (C_2_H_4_As_4_O^+^•; calcd.: 343.7126). APCI-MS (positive ion mode): *m*/*z* 391 [(M + H)^+^]; APCI-MS (positive ion mode): *m*/*z* 439 [(M + O + H + MeOH)^+^], 423 [(M + H + MeOH)^+^], 407 [(M + O + H)^+^], 391 [(M + H)^+^]; APCI-MS/MS (391): *m*/*z* 361[(M + H -CH_2_O)^+^], 345 [(M + H -CH_2_S)^+^], 285, 257; APCI-(MS)^3^: 391 → 361 → 253 → 225.

### 3.4. Computational Details

The structure of each species was optimized at the non-relativistic B3LYP/6-311G(2d,2p) level, which was previously found to be appropriate for the other arsenicals [23]. Natural bond orbital (NBO) analysis [26,27,28,29] calculations were performed with the NBO 3.0 software incorporated into Gaussian 03. For the optimized structures, the NMR parameters, i.e., nuclear shieldings (σ) and coupling constants (*J*), were calculated with three different methods. Methods A and B are non-relativistic and employed the hybrid B3LYP functional. They differ in adopting either the cc-pVTZ basis set (A) or a mixed basis set (B) comprising the pcJ-2 basis set [30] for S, O, C, and H, and cc-pVTZ for As. This choice was dictated by the good performance of the pcJ-n basis sets in the calculation of coupling constants [30,31]; however, this basis set is not available for As. Method C includes relativistic corrections by means of the zeroth-order regular approximation (ZORA) up to spin-orbit coupling (ZSO) with the Becke88-Perdew86 GGA functional (BP) and a double-zeta, twice-polarized Slater basis set (TZ2P). For the calculation of the ECD spectrum for arsenicin B, its geometry was further optimized at the B3LYP/6-311+g(3df,2pd) level, and the spectrum was calculated also at the same level utilizing time-dependent density functional theory (TD-DFT). This level of theory was previously found to be suitable in predicting qualitatively and quantitatively good electronic spectra for the structurally similar arsenicin A [20]. Geometry optimizations and non-relativistic calculations were performed with Gaussian 03 [32], and relativistic NMR parameter calculations with ADF 2007 [33] and associated NMR [34,35,36,37] and CPL [38,39] modules, which allow for the calculation of NMR properties within the ZORA. Chemical shifts were calculated from δ = σ_ref_ − σ. TMS (tetramethylsilane) was used as the reference compound, with the following σ_ref_ values for each method. ^13^C: 184.5978 (A), 181.1052 (B), 186.88 (C); ^1^H: 31.7660 (A), 31.6927 (B), 31.64 (C).

### 3.5. Antimicrobial Assays

Bacteria (*S. aureus* and *E. coli*) were grown on Mueller Hinton agar plates, and seeded after 24 h of growth at 37 °C on fresh Mueller Hinton agar plates. Candida albicans was grown on Sabouraud Agar plates (pH 6) and also seeded after 24 h of growth at 37 °C on fresh Sabouraud agar plates. Cellulose disks (6-mm diameter) impregnated with the natural compounds (see Table 6) and the gentamycin control compound were deposited on these plates. The inhibition diameters were measured after 24 h of incubation of the plates at 37 °C.

## 4. Conclusions

Following our previous report on arsenicin A, isolated from the poecilosclerid sponge *Echinochalina bargibanti* as the first polyarsenic compound in Nature, we report here new sulfurated polyarsenic metabolites, called arsenicin B and arsenicin C, which were isolated from the same sponge. These metabolites differ from the adamantane-type structure of arsenicin A by their noradamantane framework, having a different heteroatoms connectivity, and containing a very unusual As–As bond. Structure **2** for arsenicin B rests on spectral data supported by the X-ray validated structure of a recently reported synthetic product. The incomplete set of spectral data obtained for the thermally labile arsenicin C left the relative positions of oxygen and sulfur atoms unassigned. This issue was circumvented by simulating its NMR spectra with DFT calculations, including heavy-atom spin-orbit effects on ^13^C shieldings. This allowed us to assign structure **3** to arsenicin C, based on the method validated on a series of trial structures of arsenicin B. The absolute configurations of metabolites **2** and **3** were determined with a combined use of experimental and TD–DFT calculated ECD spectra, and experimentally obtained specific rotations.

Arsenicins A–C represent a new class of natural products that has no precedent. Great challenges are posed by these unique compounds. The discrimination between the biosynthesis of the arsenicins by either the sponge enzymes or microbial symbionts, as well as the identification of the biosynthetic pathways still remain unresolved. Finally, a rethinking about the recycling of arsenic in Nature must be considered after the discovery of these compounds.

The results for antimicrobial evaluation against *Staphylococcus aureus* showed that arsenicins A–C have biological effects that are similar to those of the antibiotic gentamycin. Furthermore, the promising results obtained on arsenicin A and its related synthetic compounds embolden also including also sulphurated polyarsenicals in the next studies for the development of new, eventually more potent and selective arsenical agents for therapeutic applications against cancer.

## Figures and Tables

**Table 1:** NMR spectral data for arsenicin B and arsenicin C in CDCl_3_ (^1^H at 400 MHz, ^13^C at 75 MHz, *J* in Hz). nOe: nuclear Overhauser enhancement.

C-Atom	δ_H_ (ppm), *J* (Hz)	δ_C_ (ppm)	HMBC Correlation with	nOe Enhancement at
Arsenicin B ^a^				
1	H_a_ 3.96 (*d*, 12.4) H_b_ 2.34 (*d*, 12.4)	42.20 *t*	C-2	1-H_b_ 1-H_a_
2	H_a_ 3.46 (*dd*, 13.5, 1.7) H_b_ 1.92 (*d*, 13.5)	27.40 *t*		2-H_b_ 2-H_a_
3	H_a_ 3.44 (*dd*, 13.8, 1.7) H_b_ 1.56 (*d*, 13.8)	25.70 *t*		3-H_b_ 3-H_a_
Arsenicin C				
1	H_a_ 3.50 (*d*, 12.8) H_b_ 2.02 (*dd*, 12.8, 1.7)	45.14 *t*		1-H_b_ 1-H_a_
2	H_a_ 2.99 (*dd*, 13.7, 1.9) H_b_ 1.44 (*dd*, 13.7, 1.7)	31.40 *t*	C-1	2-H_b_ 2-H_a_
3	H_a_ 3.27(*dd*, 13.8, 1.9) H_b_ 1.29 (*d*, 13.8)	33.58 *t*		3-H_b_ 3-H_a_

^a^ from Lu et al., 2015 [21]: ^1^H NMR (300 MHz, CDCl_3_): δ 1.57 (*d*, *J* 13.5), 1.93 (*d*, *J* 13.5), 2.35 (*d*, *J* 12.3) 3.45 (*dd*, *J* 13.5, 1.8), 3.47 (*dd*, *J* 13.5, 1.8 Hz), 3.97 (*d*, *J* 12.3 Hz); ^13^C NMR (126 MHz, CDCl_3_): δ 25.9, 27.4, 42.3.

**Table 2:** Experimental and calculated ^1^H and ^13^C chemical shifts (ppm) for structure **B6** of arsenicin B. C and H descriptors are given according to the numbering in Figure 1. Definitions of methods (A)–(C) are presented in Section 3.4.

Nucleus	Exptl	(A)	(B)	(C)	*σ*_SO_
C1	42.20	57.57	59.47	58.44	6.66
C2	27.40	42.57	44.05	41.40	7.37
C3	25.70	41.00	42.26	39.40	6.75
H1a	3.96	3.35	3.32	3.99	−0.37
H1b	2.34	1.98	1.96	2.41	−0.25
H2a	3.46	3.00	2.99	3.46	−0.24
H2b	1.92	1.49	1.49	2.00	−0.28
H3a	3.44	2.92	2.89	3.26	−0.30
H3b	1.56	1.15	1.15	1.49	−0.20
MAE (^13^C)		15.28	16.83	14.65	
MAE (^1^H)		0.46	0.48	0.07	
MAE (all)		5.40	5.93	4.93	
*a* (^13^C) ^a^		1.0	1.0	1.2	
*b* (^13^C) ^a^		15.0	15.5	9.8	
R(^13^C)		0.9999	0.9999	0.9999	
*a* (^1^H) ^a^		0.93	0.92	0.98	
*b* (^1^H) ^a^		−0.26	−0.25	0.05	
R(^1^H)		0.9983	0.9981	0.9948	

^a^ The correlation between the experimental and calculated values is represented by a linear equation: δ_calcd_ = aδ_exptl_ + b.

**Table 3:** Energies of trial structures **C1**–**C3** of arsenicin C by density functional theory (DFT) calculation at a B3LYP/6-311G (2d,2p) level of theory.

Structure	*E* (au)	Δ*E* (kcal/mol)
C1	−9535.03540580	1.5
C2	−9535.03778732	0.0
C3	−9535.03662772	0.7

**Table 4:** Experimental and calculated ^1^H and ^13^C chemical shifts (ppm) for the candidate structures **C1** and **C2** of arsenicin C. H descriptors 1a–3b in brackets are given according to the numbering in Figure 1. Definitions of methods (A)–(C) are reported in Section 3.4.

Nucleus	Exptl	C1				C2			
		(A)	(B)	(C)	σ_SO_	(A)	(B)	(C)	σ_SO_
C_1_	45.14	50.38	52.04	54.93	3.30	57.67	59.70	59.90	5.09
C_2_	31.40	43.25	44.90	40.87	8.90	44.16	45.84	44.27	5.96
C_3_	33.58	46.37	47.96	45.82	5.97	47.04	48.69	46.62	6.01
H1a	3.50	2.93	2.92	3.52	−0.22	2.98	2.98	3.59	−0.31
H1b	2.02	1.70	1.69	2.08	−0.23	1.60	1.62	2.25	−0.35
H2a	2.99	2.94	2.93	3.52	−0.30	2.62	2.63	3.03	−0.16
H2b	1.44	1.17	1.17	1.66	−0.31	0.96	0.99	1.65	−0.38
H3a	3.27	3.39	3.38	3.72	−0.22	2.90	2.88	3.24	−0.23
H3b	1.29	1.01	1.03	1.45	−0.20	0.78	0.80	1.38	−0.34
MAE (^13^C)		9.96	11.59	10.50		12.92	14.70	13.56	
MAE (^1^H)		0.27	0.27	0.24		0.44	0.43	0.12	
MAE (all)		3.50	4.04	3.66		4.60	5.19	4.60	
*a* (^13^C)		0.5	0.5	1.0		1.0	1.0	1.1	
*b* (^13^C)		29.7	31.3	12.5		14.3	15.1	8.4	
R (^13^C)		0.9543	0.9574	0.9788		0.9984	0.9988	0.9999	
*a* (^1^H)		1.04	1.03	1.06		1.03	1.01	0.93	
*b* (^1^H)		−0.32	−0.29	0.09		−0.51	−0.46	0.26	
*R* (^1^H)		0.9730	0.9724	0.9815		0.9978	0.9979	0.9963	

**Table 5:** Experimental and calculated *J*_H,H_ values (in Hz) for the candidate structures **C1** and **C2** of arsenicin C, according to the computational methods (A)–(C), defined as in Section 3.4.

	Exptl	C1			C2		
		(A)	(B)	(C)	(A)	(B)	(C)
^2^ *J*_H1a,H1b_	12.8	−8.73	−11.50	−6.58	−8.90	−11.65	−6.82
^2^ *J*_H2a,H2b_	13.7	−9.80	−12.69	−7.52	−9.87	−12.70	−7.69
^2^ *J*_H3a,H3b_	13.8	−9.83	−12.79	−7.65	−9.70	−12.61	−7.54
^4^ *J*_H1a,H2a_		1.52	1.27	0.60	1.61	1.46	0.74
^4^ *J*_H1a,H2b_		−0.20	−0.11	−0.29	−0.26	−0.16	−0.37
^4^ *J*_H1b,H2a_		−0.34	−0.29	−0.43	−0.21	−0.11	−0.32
^4^ *J*_H1b,H2b_	1.7	−0.38	−0.06	−0.09	0.26	0.71	0.48
^4^ *J*_H2a,H3a_	1.9	0.04	0.52	0.31	0.36	0.91	0.57
^4^ *J*_H2a,H3b_		−0.74	−0.61	−0.66	−0.37	−0.18	−0.35
^4^ *J*_H2b,H3a_		−0.80	−0.70	−0.70	−0.48	−0.32	−0.45
^4^ *J*_H2b,H3b_		1.02	0.99	0.60	1.54	1.59	0.96
^5^ *J*_H1a,H3a_		−0.08	0.15	0.26	−0.16	0.06	0.24
^5^ *J*_H1a,H3b_		−0.90	−0.83	−0.58	−0.81	−0.72	−0.47
^5^ *J*_H1b,H3a_		−0.89	−0.79	−0.51	−0.84	−0.73	−0.52
^5^ *J*_H1b,H3b_		−1.03	−0.95	−0.60	−1.03	−0.95	−0.64
MAE (*J*_H,H_) ^a^		3.1	1.3	4.4	3.0	1.1	4.2

^a^ Involves only ^2^*J*(H1a,H1b), ^2^*J*(H2a,H2b), ^2^*J*(H3a,H3b), ^4^*J*(H1b,H2b), and ^4^*J*(H2a,H3a).

**Table 6:** Antimicrobial activities reported as inhibition diameters in mm for arsenicin B (**2**) and C (**3**), in comparison with arsenicin A, and to gentamycin when used as a control test sample.

Compound	Weight	*S. aureus*	*E. coli*	*C. albicans*
Arsenicin A ^a^	10 μg/disc	24	28	26
	5 μg/disc	23	n.t.	23
	1 μg/disc	19	17	12
Arsenicin B	10 μg/disc	15	n.t. ^b^	13
	5 μg/disc	14	n.t.	12
	1 μg/disc	10	n.t.	n.t.
Arsenicin C	10 μg/disc	21	12	16
	5 μg/disc	20	n.t.	9
	1 μg/disc	10	7	n.t.
Gentamycin	10 μg/disk	22	30	22

^a^ data already reported by Mancini et al., 2006 [15]. ^b^ n.t.: not tested (compounds obtained in minute quantities).

## Supplementary Materials

The following are available online at http://www.mdpi.com/1660-3397/16/10/382/s1, Figure S1: MS spectra of arsenicin B: EI–MS spectrum and APCI(+)-MS tandem fragmentation experiment on *m*/*z* 407 [M + H]+; Figure S2: Detailed region in ^1^HNMR spectrum of arsenicin B in CDCl_3_; Figure S3: MS spectra of arsenicin C: EI–MS spectrum and APCI(+)-MS tandem fragmentation experiment on *m*/*z* 391 [M + H]+; Figure S4: Correlations between experimental and calculated ^1^H and ^13^C chemical shifts for structure **B6** of arsenicin B; Figure S5: Experimental and calculated ^1^H spectra of structure **B6** of arsenicin B; Figure S6: Theoretical ECD spectrum for the (1*R*,3*S*,5*R*,7*R*)-enantiomer of arsenicin B calculated at the TD-DFT B3LYP/6-311+G(3df,2pd) level for a structure optimized at the same level; Figure S7: Top: Correlations between the experimental and calculated (B3LYP/cc-pVTZ//B3LYP/6-311G(2d,2p) ^1^H (left) and ^13^C (right) chemical shifts for the proposed structures for arsenicin C. Bottom: Correlations between the experimental and calculated (B3LYP/cc-pVTZ//B3LYP/6-311G(2d,2p) 1H (a) 13C (b) and chemical shifts for the proposed structure **C2**; Table S1: Energies of trial structures **B1**–**B9** of arsenicin B calculated at B3LYP/6-311G(2d,2p) level; Table S2: Experimental and calculated J(^1^H,^1^H) values (in Hz) for structure **B6** of arsenicin B.
