# Supplementary material for: New Sulfur-Containing Polyarsenicals from the New Caledonian Sponge Echinochalina bargibanti
Source: Mar Drugs. 2018 Oct 11;16(10):382. doi: 10.3390/md16100382 (PMC6212947; doi:10.3390/md16100382)
Supplement: Supplementary file 1 [file marinedrugs-16-00382-s001.pdf]

Supplementary Materials

New Sulfur-Containing Polyarsenicals from the New Caledonian sponge *Echinochalina bargibanti*

Petri Tähtinen [1], Graziano Guella [2], Giacomo Saielli[3], Cécile Debitus[4], Edouard Hnawia[5] and Ines Mancini [2,*]

[1] Department of Chemistry, University of Turku, Vatselankatu 2, 20500 Turku, Finland; petri.tahtinen@utu.fi
[2] Laboratorio di Chimica Bioorganica, Dipartimento di Fisica, Università di Trento, Via Sommarive 14, I-38123 Trento, Italy; graziano.guella@unitn.it; ines.mancini@unitn.it
[3] Istituto CNR per la Tecnologia delle Membrane, Unità di Padova, and Dipartimento di Scienze Chimiche, Università di Padova, Via Marzolo, 1 - 35131, Padova, Italy; giacomo.saielli@unipd.it
[4] LEMAR, IRD, UBO, CNRS, IFREMER, IUEM, 29280 Plouzané, France;cecile.debitus@ird.fr
[5] Laboratoire Insulaire du Vivant et de l'Environnement, Université de la Nouvelle-Calédonie : EA 4243 BP 11106 98802 Nouméa, Nouvelle-Calédonie; edouard.hnawia@ird.fr

Contents

Figure S1.MS spectra Arsenicin B: EI-MS spectrum (top) and APCI(+)-MS tandem fragmentation experiment on *m/z* 407 $[M+H]^+$ (bottom).

Figure S2. Detailed region in ^{1}HNMR spectrum of arsenicin B in $CDCl_3$ (d at 1.56 ppm not shown because superimposed by water signal).

Figure S3. MS spectra of arsenicin C: EI-MS spectrum (top) and APCI(+)-MS tandem fragmentation experiment on *m/z* 391 $[M+H]^+$ (bottom).

Figure S4. Correlations between experimental and calculated ^{1}H (left) and ^{13}C (right) chemical shifts for structure **B6** of Arsenicin B. Squares: non-relativistic (method A); circles: non-relativistic (method B); triangles: relativistic (method C). The correlation line refers to method C. Details on methods in the text, par.3.4.

Figure S5. Experimental (top) and calculated (bottom) ^{1}H spectra of structure **B6** of arsenicin B. Spectrum recorded in $CDCl_3$ at 400MHz. Chemical shifts calculated with method (C), couplings with method (B). Simulations at 400 MHz. Details on methods in the text, par.3.4.

Figure S6. Theoretical ECD spectrum for the (1R,3S,5R,7R)-enantiomer of arsenicin B calculated at the TD-DFT B3LYP/6-311+G(3df,2pd) level for a structure optimized at the same level. Details on methods in the text, par.3.4.

Figure S7. Correlations between the experimental and calculated (B3LYP/cc-pVTZ//B3LYP/6-311G(2d,2p) ^{1}H (left) and ^{13}C (right) chemical shifts for the proposed structures for arsenicin C. Squares: **C1** (– –); circles: **C2** (—); triangles: **C3** (••••).

Table S1. Energies of trial structures **B1-B9** (reported in Figure 2) of arsenicin B calculated at B3LYP/6-311G(2d,2p) level.

Table S2. Experimental and calculated $J(^1H,^1H)$ values (in Hz) for structure **B6** (Figure 2) of arsenicin B.

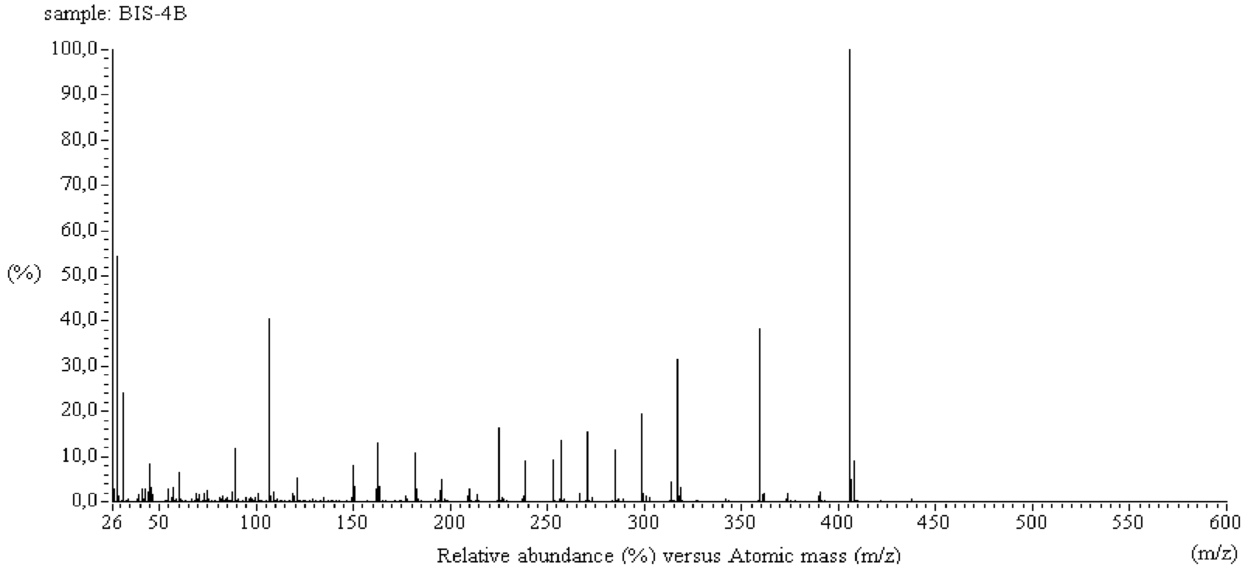

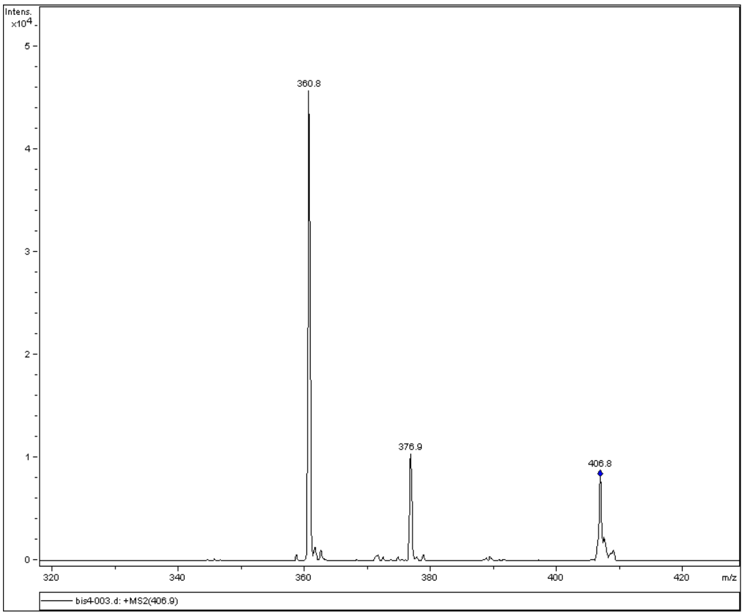

Figure S1. MS spectra of arsenicin B: EI-MS spectrum (top) and APCI(+)-MS tandem fragmentation experiment on *m*/*z* 407 $[M+H]^+$ (bottom).

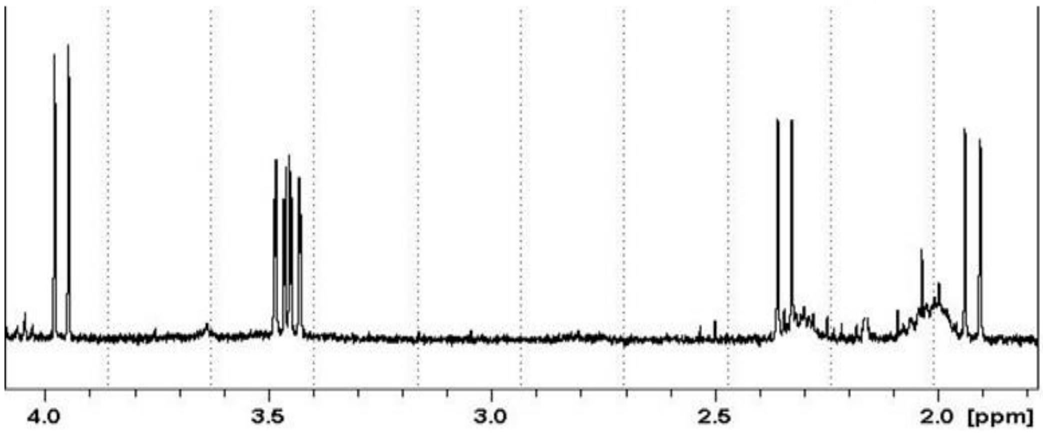

Figure S2. Detailed region in ^{1}HNMR spectrum of arsenicin B in $CDCl_3$ (d at 1.56 ppm not shown because superimposed by water signal).

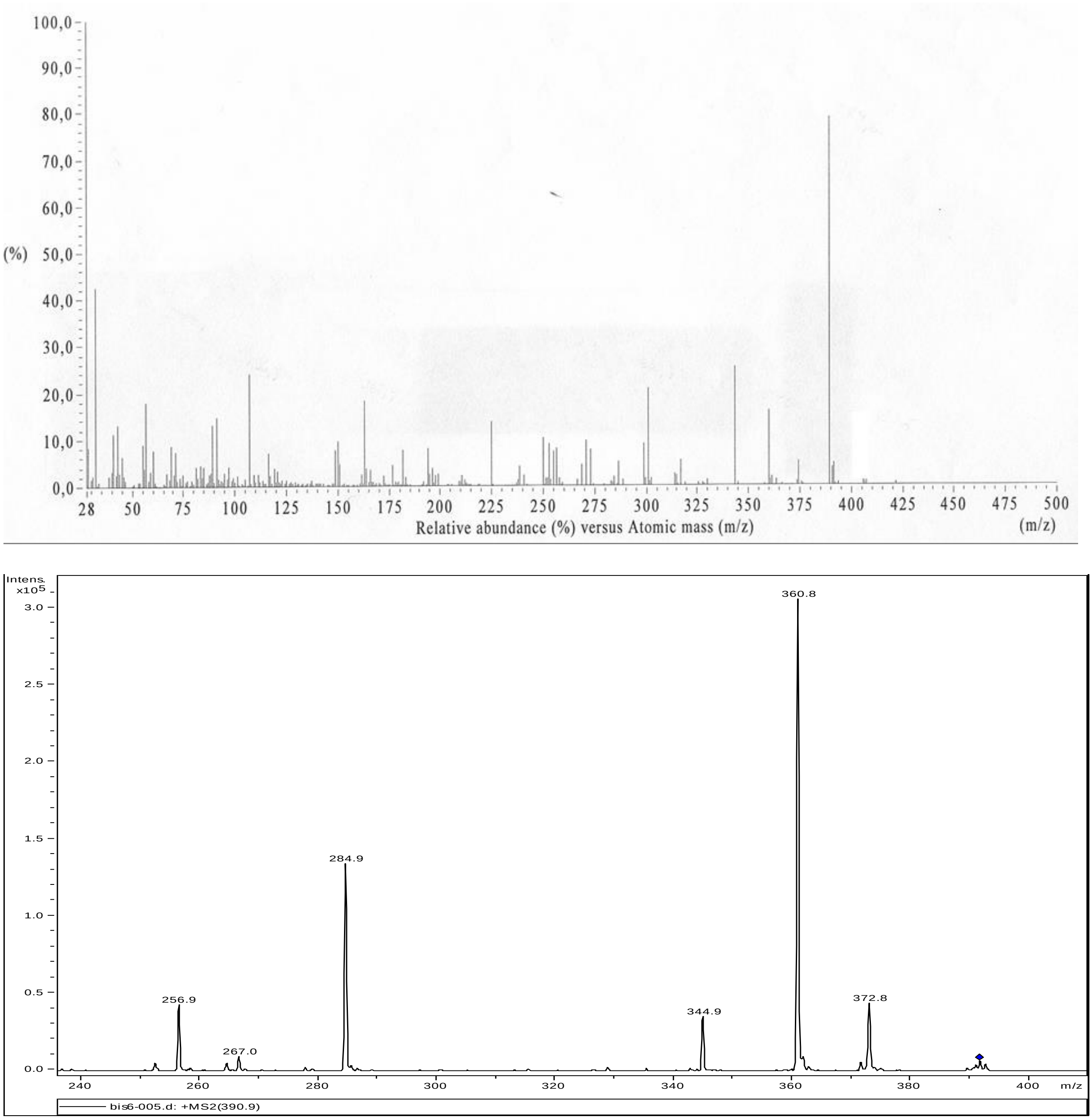

Figure S3. MS spectra of arsenicin C: EI-MS spectrum (top) and APCI(+)-MS tandem fragmentation experiment on *m/z* 391 $[M+H]^+$ (bottom).

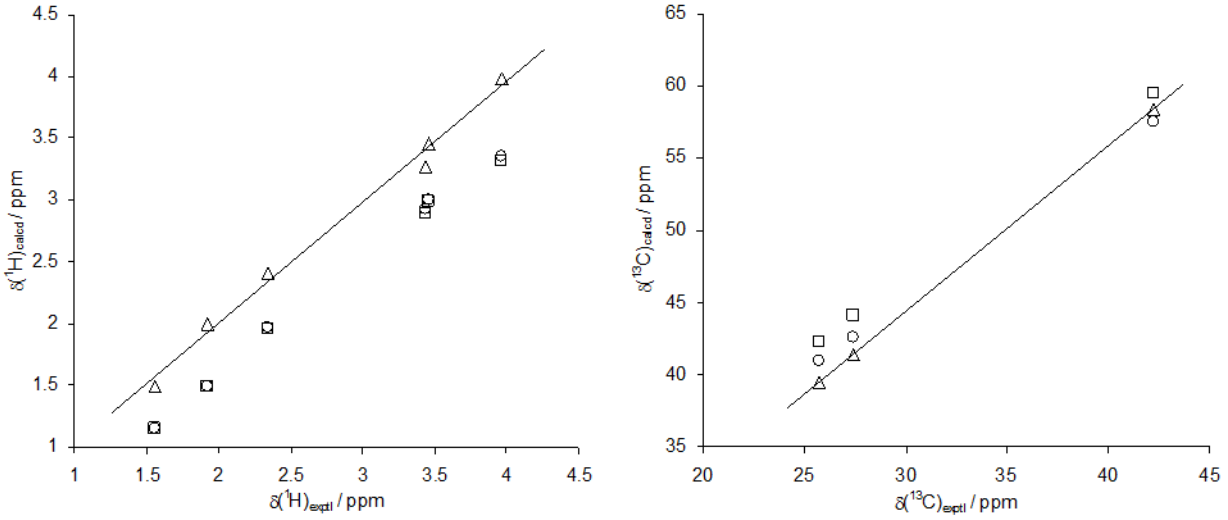

Figure S4. Correlations between experimental and calculated ^{1}H (left) and ^{13}C (right) chemical shifts for structure **B6** of Arsenicin B. Squares: non-relativistic (method A); circles: non-relativistic (method B); triangles: relativistic (method C). The correlation line refers to method C. Details on methods in the text, par.3.4.

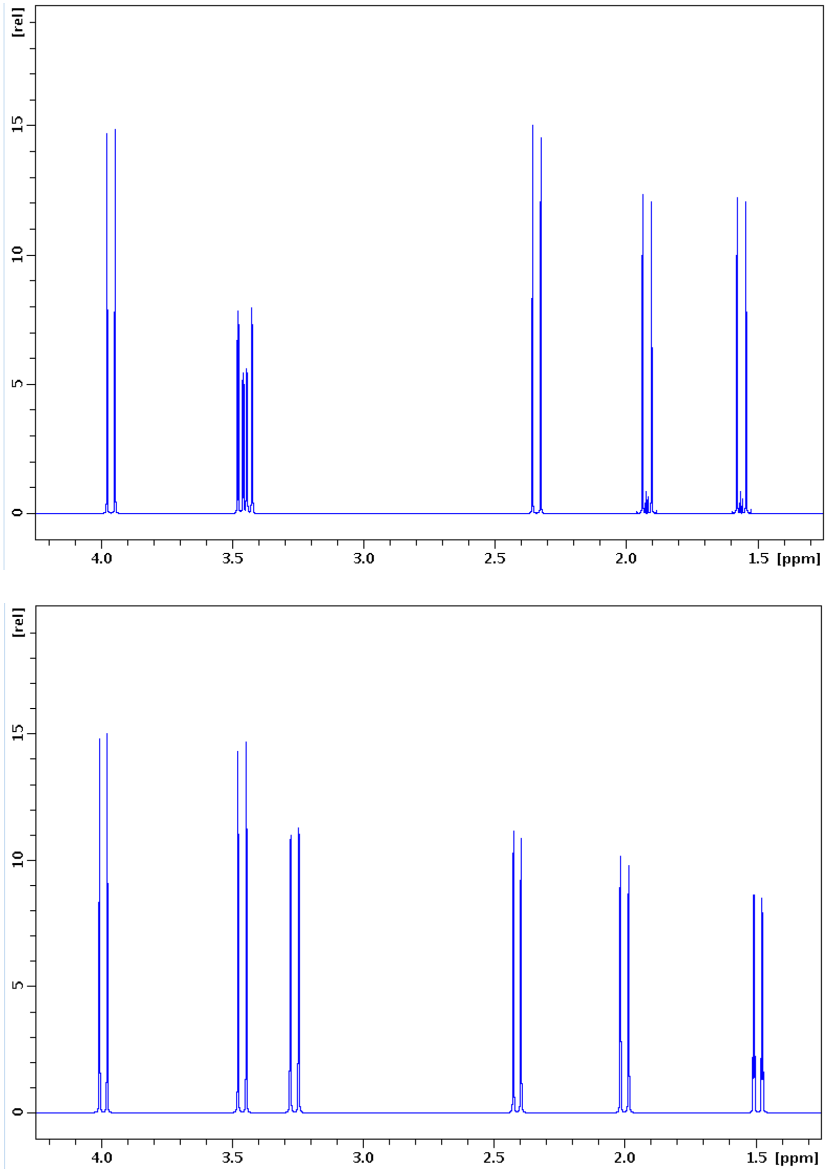

Figure S5. Experimental (top) and calculated (bottom) ^{1}H spectra of structure **B6** of arsenicin B. Spectrum recorded in $CDCl_3$ at 400MHz. Chemical shifts calculated with method (C), couplings with method (B). Simulations at 400 MHz. Details on methods in the text, par.3.4.

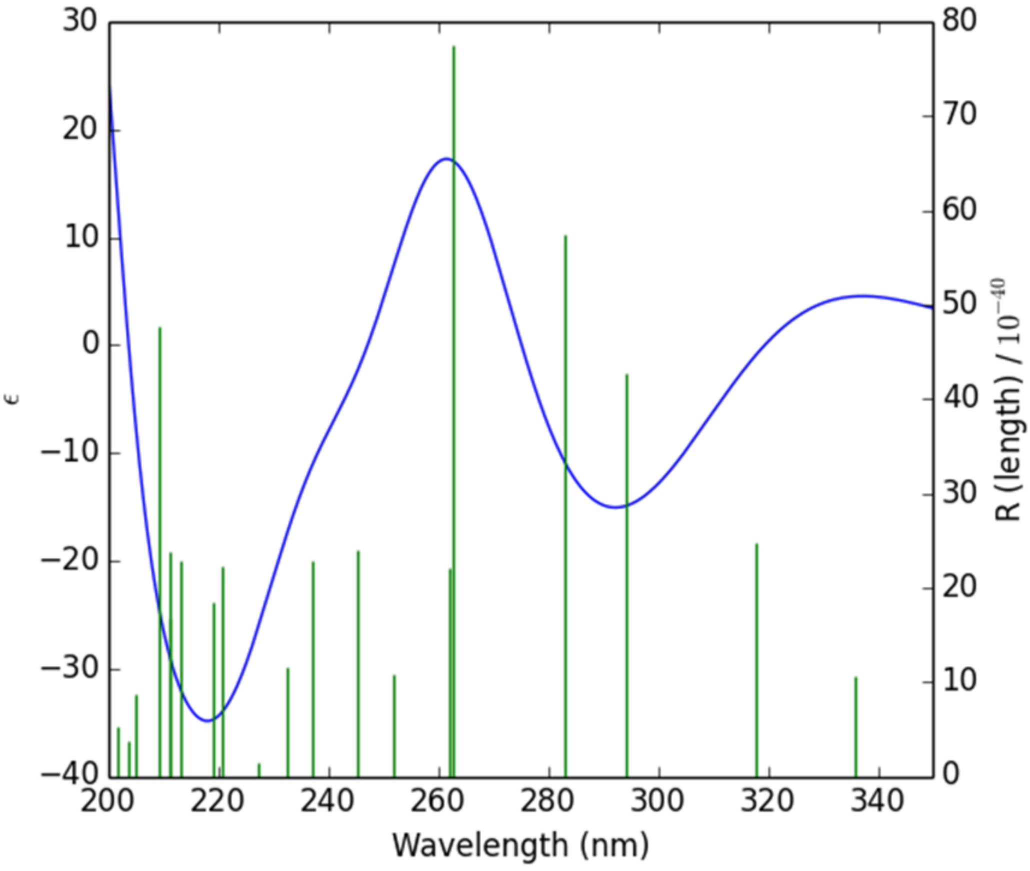

Figure S6. Theoretical ECD spectrum for the (1R,3S,5R,7R)-enantiomer of arsenicin B calculated at the TD-DFT B3LYP/6-311+G(3df,2pd) level for a structure optimized at the same level. Details on methods in the text, par.3.4.

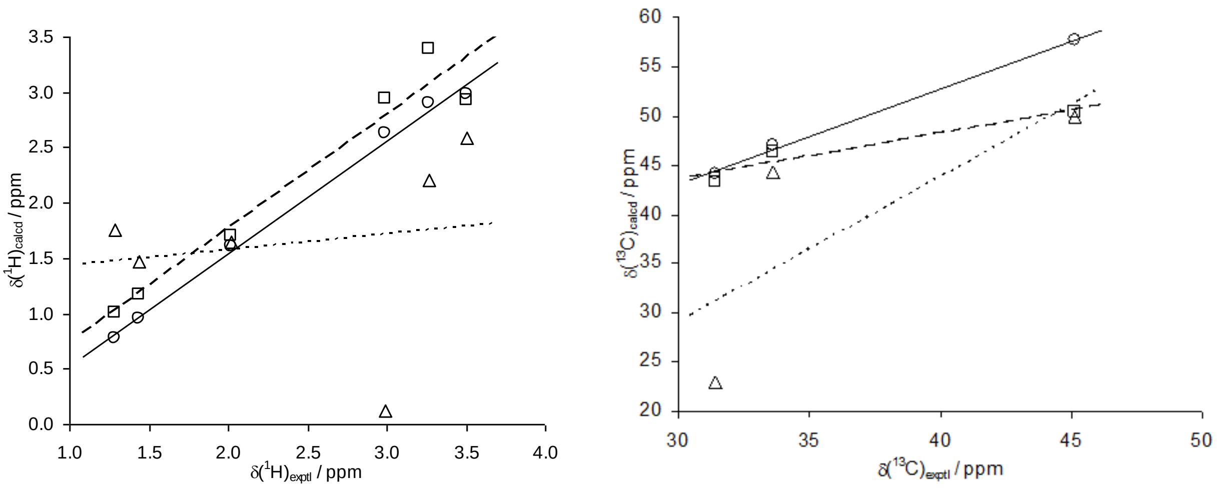

Figure S7. Correlations between the experimental and calculated (B3LYP/cc-pVTZ//B3LYP/6-311G(2d,2p) ^{1}H (left) and ^{13}C (right) chemical shifts for the proposed structures for arsenicin C. Squares: **C1** (– –); circles: **C2** (—); triangles: **C3** (····).

Table S1. Energies of trial structures **B1-B9** (reported in Figure 2) of arsenicin B calculated at B3LYP/6-311G(2d,2p) level.

**Structure**	**Symmetry group**	***E* (au)**	**Δ*E* (kcal/mol)**
**B1**	C_1	-9857.86618198	316
**B2'**	C_1	-9857.92232084	281
**B3**	C_1	-9858.02119547	219
**B4**	C_1	-9858.02232713	218
**B5**	C_1	-9858.02109254	219
**B6**	C_1	-9858.36900792	0.5
**B7**	C_s	-9858.36937360	0.2
**B8**	C_s	-9858.36765298	1.3
**B9**	C_2	-9858.36972534	(0.0)

Table S2. Experimental and calculated $J(^1H,^1H)$ values (in Hz) for structure **B6** (Figure 2) of arsenicin B.

	**Exptl** [a]	**(A)**	**(B)**	**(C)**
^2J(1a,1b)	12.4	-8.6	-11.4	-6.4
^2J(2a,2b)	13.5	-9.7	-12.6	-7.5
^2J(3a,3b)	13.8	-9.7	-12.7	-7.6
^4J(1a,2a)		1.6	1.4	0.7
^4J(1a,2b)		-0.3	-0.2	-0.4
^4J(1b,2a)		-0.3	-0.2	-0.4
^4J(1b,2b)		-0.5	-0.2	-0.2
^4J(2a,3a)	1.7	0.2	0.7	0.5
^4J(2a,3b)		-0.8	-0.7	-0.7
^4J(2b,3a)		-0.8	-0.7	-0.7
^4J(2b,3b)		1.0	0.9	0.6
^5J(1a,3a)		-0.2	0.0	0.1
^5J(1a,3b)		-0.8	-0.7	-0.5
^5J(1b,3a)		-0.8	-0.7	-0.5
^5J(1b,3b)		-1.0	-0.9	-0.6
MAE(J_{HH}) [b]		3.3	1.0	4.9

[a]Experimental data are absolute values.

[b] Involves only ^2J(H7,H8), ^2J(H9,H10), ^2J(H13,H14) and ^4J(H7,H13).
